# Supplementary material for: Thresholds for adding degraded tropical forest to the conservation estate
Source: Nature. 2024 Jul 17;631(8022):808–13. doi: 10.1038/s41586-024-07657-w (PMC11269177; doi:10.1038/s41586-024-07657-w)
Supplement: Supplementary file 1 — Supplementary Table 1, providing details of the datasets we combined for these analyses. Supplementary Table 2, listing the functional groups we analysed. [file 41586_2024_7657_MOESM1_ESM.pdf]

nature portfolio

https://doi.org/10.1038/s41586-024-07657-w

# Supplementary information

# Thresholds for adding degraded tropical forest to the conservation estate

In the format provided by the authors and unedited

# Thresholds for adding degraded tropical forest to the conservation estate

Robert M. EWERS; C. David L. ORME; William D. PEARSE; Nursyamin ZULKIFLI; Genevieve YVON-DUROCHER; Kalsum M. YUSAH; Natalie YOH; Darren C. J. YEO; Anna WONG; Joseph WILLIAMSON; Clare L WILKINSON; Fabienne WIEDERKEHR; Bruce L. WEBBER; Oliver R. WEARN; Leona WAI; Maisie VOLLANS; Joshua P. TWINING; Edgar C. TURNER; Joseph A. TOBIAS; Jack THORLEY; Elizabeth M. TELFORD; Yit Arn TEH; Heok Hui TAN; Tom SWINFIELD; Martin SVÁTEK; Matthew STRUEBIG; Nigel STORK; Jani SLEUTEL; Eleanor M. SLADE; Adam SHARP; Adi SHABRANI; Sarab S. SETHI; Dave J. I. SEAMAN; Anati SAWANG; Gabrielle Briana ROXBY; J. Marcus ROWCLIFFE; Stephen J. ROSSITER; Terhi RIUTTA; Homathevi RAHMAN; Lan QIE; Elizabeth PSOMAS; Aaron PRAIRIE; Frederica POZNANSKY; Rajeev PILLAY; Lorenzo PICINALI; Annabel PIANZIN; Marion PFEIFER; Jonathan M. PARRETT; Ciar D. NOBLE; Reuben NILUS; Nazirah MUSTAFFA; Katherine E. MULLIN; Simon MITCHELL; Amelia R. MCKINLAY; Sarah MAUNSELL; Radim MATULA; Michael MASSAM; Stephanie MARTIN; Yadvinder MALHI; Noreen MAJALAP; Catherine S. MACLEAN; Emma MACKINTOSH; Sarah H. LUKE; Owen T. LEWIS; Harry J. LAYFIELD; Isolde LANE-SHAW; Boon Hee KUEH; Pavel KRATINA; Oliver KONOPIK; Roger KITCHING; Lois KINNEEN; Victoria A. KEMP; Palasiah JOTAN; Nick JONES; Evyen W. JEBRAIL; Michal HRONEŠ; Sui Peng HEON; David R. HEMPRICH-BENNETT; Jessica K. HAYSOM; Martina F. HARIANJA; Jane HARDWICK; Nichar GREGORY; Ryan GRAY; Ross E. J. GRAY; Natasha GRANVILLE; Richard GILL; Adam FRASER; William A. FOSTER; Hollie FOLKARD-TAPP; Robert J. FLETCHER; Arman Hadi FIKRI; Tom M. FAYLE; Aisyah FARUK; Paul EGGLETON; David P. EDWARDS; Rosie DRINKWATER; Rory A. DOW; Timm F. DÖBERT; Raphael K. DIDHAM; Katharine J. M. DICKINSON; Nicolas J. DEERE; Tijmen DE LORM; Mahadimenakbar M. DAWOOD; Charles W. DAVISON; Zoe G. DAVIES; Richard G. DAVIES; Martin DANČÁK; Jeremy CUSACK; Elizabeth L. CLARE; Arthur CHUNG; Vun Khen CHEY; Philip M. CHAPMAN; Lauren CATOR; Daniel CARPENTER; Chris CARBONE; Kerry CALLOWAY; Emma R. BUSH; David F.R.P. BURSLEM; Keiron D. BROWN; Stephen J. BROOKS; Ella BRASINGTON; Hayley BRANT; Michael J. W. BOYLE; Sabine BOTH; Joshua BLACKMAN; Tom R. BISHOP; Jake E. BICKNELL; Henry BERNARD; Saloni BASRUR; Maxwell V. L. BARCLAY; Holly BARCLAY; Georgina ATTON; Marc ANCRENAZ; David C. ALDRIDGE; Olivia Z. DANIEL; Glen REYNOLDS; Cristina BANKS-LEITE

# Supporting information

**Supplementary Table 1. List of data sources compiled for analysis.** For each data source, we present the surname of the first author and a survey citation; a weblink to a data publication or, if that is unavailable, then a weblink to a published paper presenting the data; the types of taxa included; the number of taxa; the number of sampling methods used; the number of sampling periods; and the final number of surveys we extracted from that data source.

| First author | Link | Taxon type(s) | No. taxa | No. sampling methods | No. sample periods | No. surveys |
|---|---|---|---|---|---|---|
| Bernard [70] | https://zenodo.org/record/3908128 | mammal | 23 | 1 | 1 | 1 |
| Bishop [69] | https://zenodo.org/record/1198839 | invertebrate | 299 | 1 | 1 | 1 |
| Both [68] | https://zenodo.org/record/3247631 | plant | 262 | 1 | 1 | 1 |
| Brant [71] | https://zenodo.org/record/1198846 | invertebrate | 43 | 2 | 2 | 3 |
| Carpenter [72] | https://zenodo.org/record/5562260 | invertebrate | 106 | 6 | 1 | 6 |
| Chapman [73] | https://zenodo.org/record/2579792 | mammal | 12 | 1 | 1 | 1 |
| Deere [74] | https://zenodo.org/record/4010757 | mammal | 28 | 1 | 3 | 3 |
| Döbert [75] | https://zenodo.org/record/2536270 | plant | 1,235 | 1 | 1 | 1 |
| Drinkwater [76] | https://zenodo.org/record/3476542 | invertebrate | 2 | 1 | 2 | 2 |
| Ewers [77] | https://zenodo.org/record/3975973 | invertebrate | 17 | 1 | 1 | 1 |
| Faruk [78] | https://zenodo.org/record/1303010 | amphibian | 2 | 1 | 1 | 1 |
| Fayle [79] | https://zenodo.org/record/3876227 | invertebrate | 271 | 1 | 1 | 1 |
| Fraser [80] | https://zenodo.org/record/3973551 | amphibian | 22 | 1 | 1 | 1 |
| Fraser [81] | https://zenodo.org/record/3981222 | bird | 82 | 1 | 1 | 1 |
| Gray [82] | https://zenodo.org/record/1198302 | invertebrate | 56 | 1 | 1 | 1 |
| Gray [83] | https://zenodo.org/record/3475406 | invertebrate | 11 | 1 | 1 | 2 |
| Gregory [84] | https://zenodo.org/record/3994260 | invertebrate | 1 | 1 | 1 | 1 |
| Hardwick [85] | https://zenodo.org/record/4275386 | invertebrate | 90 | 1 | 1 | 1 |
| Hemprich-Bennett [86] | https://zenodo.org/record/3247465 | mammal | 47 | 1 | 3 | 3 |
| Heon [87] | https://doi.org/10.1890/15-1363 | mammal; bird; reptile | 62 | 1 | 6 | 6 |
| Heon [88] | https://zenodo.org/record/3955050 | mammal; reptile; bird | 35 | 1 | 7 | 7 |
| Heon [89] | https://zenodo.org/record/1304117 | mammal | 8 | 1 | 1 | 1 |
| Jebrail [90] | https://zenodo.org/record/3475408 | invertebrate | 7 | 1 | 1 | 1 |
| Kendall [91] | https://zenodo.org/record/1237736 | invertebrate | 1 | 1 | 1 | 1 |
| Konopik [92] | https://zenodo.org/record/1995439 | amphibian | 29 | 1 | 3 | 3 |
| Lane Shaw [93] | https://zenodo.org/record/1237732 | invertebrate | 21 | 1 | 1 | 1 |
| Layfield [94] | https://zenodo.org/record/1198475 | mammal | 1 | 1 | 1 | 1 |
| Luke [95] | https://zenodo.org/record/5710509 | invertebrate | 191 | 3 | 2 | 6 |
| Luke [96] | https://zenodo.org/record/1198833 | invertebrate | 27 | 2 | 1 | 2 |
| Mackintosh [97] | https://zenodo.org/record/4630980 | invertebrate | 17 | 1 | 1 | 1 |
| Maunsell [98] | https://zenodo.org/record/4247169 | invertebrate | 599 | 1 | 1 | 1 |

| | | | | | | |
|---|---|---|---|---|---|---|
| Maunsell [99] | https://zenodo.org/record/4139685 | invertebrate | 216 | 1 | 1 | 1 |
| Mitchell [100] | https://doi.org/10.1016/j.ecolind.2020.106717 | bird | 135 | 1 | 5 | 5 |
| Mullin [101] | https://zenodo.org/record/3971012 | mammal | 7 | 1 | 2 | 3 |
| Noble [102] | https://zenodo.org/record/3485086 | amphibian | 11 | 1 | 1 | 1 |
| Pianzin [103] | https://zenodo.org/record/3897377 | mammal | 2 | 1 | 1 | 1 |
| Pillay [104] | https://zenodo.org/record/3366104 | bird | 7 | 1 | 2 | 2 |
| Psomas [105] | https://zenodo.org/record/1400562 | invertebrate | 42 | 1 | 1 | 1 |
| Qie [106] | https://zenodo.org/record/3901735 | mammal; bird; reptile; invertebrate | 54 | 1 | 1 | 1 |
| Qie [107] | https://zenodo.org/record/1400564 | plant | 312 | 1 | 3 | 3 |
| Sawang [108] | https://zenodo.org/record/3354068 | invertebrate | 8 | 1 | 2 | 2 |
| Seaman [109] | https://zenodo.org/record/5109892 | mammal | 1 | 1 | 1 | 1 |
| Sethi [110] | https://zenodo.org/record/3997172 | bird; amphibian | 290 | 1 | 2 | 3 |
| Shapiro [111] | https://zenodo.org/record/1237720 | invertebrate | 10 | 1 | 1 | 1 |
| Sharp [112] | https://zenodo.org/record/1323504 | invertebrate | 594 | 1 | 2 | 11 |
| Slade [113-115] | https://zenodo.org/record/3247492 https://zenodo.org/record/3247494 https://zenodo.org/record/3832076 | invertebrate | 83 | 1 | 3 | 3 |
| Slade [116,117] | https://zenodo.org/record/3906118 https://zenodo.org/record/3906441 | invertebrate | 72 | 1 | 2 | 2 |
| Turner [118] | https://zenodo.org/record/5729342 | plant | 123 | 1 | 10 | 10 |
| Twining [119] | https://zenodo.org/record/1237731 | reptile; mammal | 6 | 1 | 1 | 1 |
| Vollans [120] | https://zenodo.org/record/3929764 | invertebrate | 2 | 1 | 1 | 1 |
| Wilkinson [121] | https://zenodo.org/record/4072959 | fish | 36 | 3 | 5 | 10 |
| Williamson [122] | https://zenodo.org/record/1487595 | invertebrate | 15 | 1 | 1 | 1 |

**Supplementary Table 2. List of functional groups used in analyses.** Groups are a combination of up to four factors: (1) Category – used to aggregate functional groups into sets of related functions; (2) Taxon – used to separate functional groups by taxonomic identity; (3) Attributes – specific morphology, behaviour, life history strategy or conservation status; and (4) Level – used to separate attributes by categories of increasing value.

| Category | Taxon[a] | Attributes | Level |
|---|---|---|---|
| **Body mass** | insect | | low |
| **Body mass** | insect | | medium |
| **Body mass** | insect | | high |
| **Body mass** | ant | | low |
| **Body mass** | ant | | medium |
| **Body mass** | ant | | high |
| **Body mass** | mammal | | low |
| **Body mass** | mammal | | medium |
| **Body mass** | mammal | | high |
| **Body mass** | bird | | low |
| **Body mass** | bird | | medium |
| **Body mass** | bird | | high |
| **Body mass** | amphibian | | low |
| **Body mass** | amphibian | | medium |
| **Body mass** | amphibian | | high |
| **Body mass** | fish | | low |
| **Body mass** | fish | | medium |
| **Body mass** | fish | | high |
| **Trophic** | all taxa | parasitoid | |
| **Trophic** | all taxa | parasite | |
| **Trophic** | all taxa | carnivore | |
| **Trophic** | all taxa | herbivore | |
| **Trophic** | all taxa | saprophage | |
| **Trophic** | all taxa | producer | |
| **Trophic** | all taxa | generalism | low |
| **Trophic** | all taxa | generalism | high[c] |
| **Diet** | all taxa | hematophage | |
| **Diet** | all taxa | piscivore | |
| **Diet** | all taxa | vertivore | |
| **Diet** | all taxa | invertivore | |
| **Diet** | all taxa | bacteriophage | |
| **Diet** | all taxa | frugivore | |
| **Diet** | all taxa | granivore | |
| **Diet** | all taxa | florivore | |
| **Diet** | all taxa | nectarivore | |
| **Diet** | all taxa | palynivore | |
| **Diet** | all taxa | folivore | |
| **Diet** | all taxa | phloeophage | |
| **Diet** | all taxa | xylophage | |

| | | | |
|---|---|---|---|
| **Diet** | all taxa | rhizophage | |
| **Diet** | all taxa | algivore | |
| **Diet** | all taxa | mycophage | |
| **Diet** | all taxa | saprophage | |
| **Diet** | all taxa | coprophage | |
| **Diet** | all taxa | necrophage | |
| **Diet** | all taxa | detritivore | |
| **Diet** | all taxa | saproxylic | |
| **Diet** | all taxa | soilphage | |
| **Diet** | all taxa | generalism | low[b] |
| **Diet** | all taxa | generalism | high[c] |
| **Movement** | invertebrate | legged | |
| **Movement** | invertebrate | legless | |
| **Movement** | insect | winged | |
| **Movement** | insect | legged | |
| **Movement** | mammal | winged | |
| **Movement** | mammal | legged | |
| **Movement** | reptile | legged | |
| **Movement** | reptile | legless | |
| **Movement** | all taxa | winged | |
| **Movement** | all taxa | legged | |
| **Movement** | all taxa | legless | |
| **Sociality** | insect | eusocial | |
| **Sociality** | insect | social | |
| **Sociality** | insect | solitary | |
| **Sociality** | mammal | social | |
| **Sociality** | mammal | pair | |
| **Sociality** | mammal | solitary | |
| **Sociality** | bird | pair | |
| **Sociality** | bird | solitary | |
| **Sociality** | amphibian | social | |
| **Sociality** | amphibian | solitary | |
| **Sociality** | all taxa | eusocial | |
| **Sociality** | all taxa | social | |
| **Sociality** | all taxa | pair | |
| **Sociality** | all taxa | solitary | |
| **Development** | invertebrate | direct | |
| **Development** | invertebrate | indirect | |
| **Development** | insect | direct | |
| **Development** | insect | indirect | |
| **Development** | all taxa | direct | |
| **Development** | all taxa | indirect | |
| **Physiology** | all taxa | endotherm | |
| **Physiology** | all taxa | ectotherm | |
| **Habitat strata**[d] | insect | arboreal | |
| **Habitat strata** | insect | understorey | |
| **Habitat strata** | insect | terrestrial | |
| **Habitat strata** | insect | subterranean | |

| | | | |
|---|---|---|---|
| **Habitat strata** | insect | aquatic | |
| **Habitat strata** | mammal | aerial | |
| **Habitat strata** | mammal | arboreal | |
| **Habitat strata** | mammal | understorey | |
| **Habitat strata** | mammal | terrestrial | |
| **Habitat strata** | mammal | subterranean | |
| **Habitat strata** | mammal | aquatic | |
| **Habitat strata** | bird | aerial | |
| **Habitat strata** | bird | arboreal | |
| **Habitat strata** | bird | understorey | |
| **Habitat strata** | bird | terrestrial | |
| **Habitat strata** | bird | aquatic | |
| **Habitat strata** | reptile | arboreal | |
| **Habitat strata** | reptile | terrestrial | |
| **Habitat strata** | reptile | subterranean | |
| **Habitat strata** | reptile | aquatic | |
| **Habitat strata** | amphibian | arboreal | |
| **Habitat strata** | amphibian | terrestrial | |
| **Habitat strata** | amphibian | subterranean | |
| **Habitat strata** | amphibian | aquatic | |
| **Habitat strata** | all taxa | aerial | |
| **Habitat strata** | all taxa | arboreal | |
| **Habitat strata** | all taxa | understorey | |
| **Habitat strata** | all taxa | terrestrial | |
| **Habitat strata** | all taxa | subterranean | |
| **Habitat strata** | all taxa | aquatic | |
| **Habitat strata** | all taxa | constant | |
| **Habitat strata** | all taxa | variable | |
| **Habitat strata** | all taxa | generalism | low[b] |
| **Habitat strata** | all taxa | generalism | high |
| **Plant** | plant | wood density | low |
| **Plant** | plant | wood density | high |
| **Plant** | plant | photosynthesis | low |
| **Plant** | plant | photosynthesis | high |
| **Plant** | plant | competitor | low |
| **Plant** | plant | competitor | high |
| **Plant** | plant | stress | low |
| **Plant** | plant | stress | high |
| **Plant** | plant | ruderal | low |
| **Plant** | plant | ruderal | high |
| **Red List status** | all taxa | threatened | |
| **Red List status** | all taxa | not threatened | |

[a] *Taxonomic groups for invertebrates have a partially nested structure. The group 'invertebrate' contains all invertebrate taxa except those belonging to class Insecta and class Arachnida. The group 'insect' contains all insect taxa (class Insecta) except ants (family Formicidae).*

[b] *Low generalism is equivalent to high specialism*

[c] *High trophic and diet generalism is equivalent to high omnivory*

[d] *Habitat strata are defined as the forest layers where a taxon forages for food*
