# Supplementary material for: Thresholds for adding degraded tropical forest to the conservation estate
Source: Nature. 2024 Jul 17;631(8022):808–13. doi: 10.1038/s41586-024-07657-w (PMC11269177; doi:10.1038/s41586-024-07657-w)
Supplement: Supplementary file 2 — Reporting Summary [file 41586_2024_7657_MOESM2_ESM.pdf]

nature portfolio

Corresponding author(s): Ewers

Last updated by author(s): May 19, 2023

Reporting Summary

Nature Portfolio wishes to improve the reproducibility of the work that we publish. This form provides structure for consistency and transparency in reporting. For further information on Nature Portfolio policies, see our Editorial Policies and the Editorial Policy Checklist.

Statistics

For all statistical analyses, confirm that the following items are present in the figure legend, table legend, main text, or Methods section.

n/a	Confirmed	
☐	☒	The exact sample size (*n*) for each experimental group/condition, given as a discrete number and unit of measurement
☐	☒	A statement on whether measurements were taken from distinct samples or whether the same sample was measured repeatedly
☐	☒	The statistical test(s) used AND whether they are one- or two-sided<br>*Only common tests should be described solely by name; describe more complex techniques in the Methods section.*
☐	☒	A description of all covariates tested
☐	☒	A description of any assumptions or corrections, such as tests of normality and adjustment for multiple comparisons
☐	☒	A full description of the statistical parameters including central tendency (e.g. means) or other basic estimates (e.g. regression coefficient) AND variation (e.g. standard deviation) or associated estimates of uncertainty (e.g. confidence intervals)
☐	☒	For null hypothesis testing, the test statistic (e.g. *F*, *t*, *r*) with confidence intervals, effect sizes, degrees of freedom and *P* value noted<br>*Give P values as exact values whenever suitable.*
☒	☐	For Bayesian analysis, information on the choice of priors and Markov chain Monte Carlo settings
☒	☐	For hierarchical and complex designs, identification of the appropriate level for tests and full reporting of outcomes
☐	☒	Estimates of effect sizes (e.g. Cohen's *d*, Pearson's *r*), indicating how they were calculated

Our web collection on statistics for biologists contains articles on many of the points above.

Software and code

Policy information about availability of computer code

Data collection	Data were compiled from online databases using the safedata R package (v 1.1.3)
Data analysis	All data manipulation, data analysis and construction of figures were conducted in the R v4.02 computing environment, using the packages ape v5.0, betareg v3.1-4, dplyr v1.1.4, lme4 v1.1-35.1, lmtest v0.9-40, lubridate v1.9.3, MASS v7.3-60.0.1, openxlsx v4.2.5.2, paletteer v1.6.0, pastecs v1.4.2, png v0.1-8, raster v3.6-26, reshape2 v1.4.4, rgdal v1.6-7, rgeos v0.6-4, safedata v1.1.3, scales v1.3.0, sf v1.0-15, spgwr v0.6-36, stringr v1.5.1 and strucchange v1.5-3.

For manuscripts utilizing custom algorithms or software that are central to the research but not yet described in published literature, software must be made available to editors and reviewers. We strongly encourage code deposition in a community repository (e.g. GitHub). See the Nature Portfolio guidelines for submitting code & software for further information.

Data

Policy information about availability of data

All manuscripts must include a data availability statement. This statement should provide the following information, where applicable:

- Accession codes, unique identifiers, or web links for publicly available datasets
- A description of any restrictions on data availability
- For clinical datasets or third party data, please ensure that the statement adheres to our policy

Datasets used in these analyses were published separately. Citations are provided in Table S1.

April 2023

Research involving human participants, their data, or biological material

Policy information about studies with human participants or human data. See also policy information about sex, gender (identity/presentation), and sexual orientation and race, ethnicity and racism.

Reporting on sex and gender	NA
Reporting on race, ethnicity, or other socially relevant groupings	NA
Population characteristics	NA
Recruitment	NA
Ethics oversight	NA

Note that full information on the approval of the study protocol must also be provided in the manuscript.

Field-specific reporting

Please select the one below that is the best fit for your research. If you are not sure, read the appropriate sections before making your selection.

☐ Life sciences ☐ Behavioural & social sciences ☒ Ecological, evolutionary & environmental sciences

For a reference copy of the document with all sections, see nature.com/documents/nr-reporting-summary-flat.pdf

Ecological, evolutionary & environmental sciences study design

All studies must disclose on these points even when the disclosure is negative.

Study description	This is a meta-analysis that combines data from 127 separate field surveys, each of which examined the distribution of organisms along a gradient of forest degradation.
Research sample	The sample is 127 individual datasets, each of which had a bespoke number of sample sites and replicates.
Sampling strategy	We sampled all datasets published on the SAFE Project Zenodo community
Data collection	Datasets used in this meta-analysis were compiled from online sources. Original field data were collected using a wide variety of methods according to the focus of the particular studies. All authors of the original data are included as authors on the manuscript.
Timing and spatial scale	All original datasets used in this meta-analysis were collected between 2010-2020 from the SAFE Project study site in Malaysia, which has a spatial extent of approximately 10,000 ha.
Data exclusions	No data were excluded
Reproducibility	Many of the individual taxa we analyse were detected in more than one field survey, meaning our analyses represent taxon-level responses that are averaged across multiple surveys.
Randomization	Datasets were grouped according to whether or not they had shared taxa in common. No other groupings of data were used.
Blinding	NA

Did the study involve field work? ☒ Yes ☐ No

Field work, collection and transport

Field conditions	Average annual rainfall at the site is ~3060 mm, and average annual temperature is ~23 degrees Celcius.
Location	The study site is located at roughly 116 degrees East 4 degrees North. The average altitude of sampling points is ~400 masl.
Access & import/export	Original datasets used in this meta-analysis were collected buy authors in line with Malaysian requirements for research in the state of Sabah. This included working productively with local collaborators, obtaining permissions from land owners, and obtaining research permission from the Sabah Biodiversity Centre.

Disturbance	Original studies used in this meta-analysis were collected using a wide variety of non-invasive and invasive survey techniques. Each was appropriate and proportional to the data being collected, and none generated lasting disturbance to the ecosystem.

Reporting for specific materials, systems and methods

We require information from authors about some types of materials, experimental systems and methods used in many studies. Here, indicate whether each material, system or method listed is relevant to your study. If you are not sure if a list item applies to your research, read the appropriate section before selecting a response.

Materials & experimental systems

n/a	Involved in the study
☒	☐ Antibodies
☒	☐ Eukaryotic cell lines
☒	☐ Palaeontology and archaeology
☒	☐ Animals and other organisms
☒	☐ Clinical data
☒	☐ Dual use research of concern
☒	☐ Plants

Methods

n/a	Involved in the study
☒	☐ ChIP-seq
☒	☐ Flow cytometry
☒	☐ MRI-based neuroimaging
